# Genetic spectrum of dyschromatosis symmetrica hereditaria in Chinese patients including a novel nonstop mutation in *ADAR1* gene

**DOI:** 10.1186/s12881-015-0255-1

**Published:** 2016-02-18

**Authors:** Guolong Zhang, Minhua Shao, Zhixiu Li, Yong Gu, Xufeng Du, Xiuli Wang, Ming Li

**Affiliations:** Department of Phototherapy at Shanghai Skin Disease Hospital & Institute of Photomedicine, Tongji University School of Medicine, 1278, Baode Road, Shanghai, 200443 China; Department of Dermatology, Nanjing Medical University, Affiliated Wuxi People’s Hospital, Wuxi, 214023 China; University of Queensland Diamantina Institute, Translational Research Institute, Brisbane, Queensland Australia; Department of Dermatology, Xinhua Hospital, Shanghai Jiaotong University School of Medicine, 1665, Kongjiang Road, Shanghai, 200092 China

**Keywords:** Dyschromatosis symmetrica hereditaria, Mutation, Nonstop, ADAR1

## Abstract

**Background:**

Dyschromatosis symmetrica hereditaria (DSH) is a rare autosomal dominant cutaneous disorder caused by the mutations of adenosine deaminase acting on RNA1 (*ADAR1*) gene. We present a clinical and genetic study of seven unrelated families and two sporadic cases with DSH for mutations in the full coding sequence of *ADAR1* gene.

**Methods:**

*ADAR1* gene was sequenced in seven unrelated families and two sporadic cases with DSH and 120 controls. Functional significance of the observed *ADAR1* mutations was analyzed using PolyPhen 2, SIFT and DDIG-in.

**Results:**

We describe six novel mutations of the *ADAR1* gene in Chinese patients with DSH including a nonstop mutation p.Stop1227R, which was firstly reported in *ADAR1* gene. In silico analysis proves that all the mutations reported here are pathogenic.

**Conclusion:**

This study is useful for functional studies of the protein and to define a diagnostic strategy for mutation screening of the *ADAR1* gene. A three-generation family exhibiting phenotypic variability with a single germline *ADAR1* mutation suggests that chilblain might aggravate the clinical phenotypes of DSH.

## Background

Dyschromatosis symmetrica hereditaria (DSH; Mendelian Inheritance in Man no. 127400) is a rare pigmentary genodermatosis of autosomal dominant inheritance with high penetrance [1]. Ethnic background appears to be a major influence on the incidence of this disorder, as it is much more commonly found in Japanese and Chinese populations than in others [2]. RNA-specific adenosine deaminase 1 (*ADAR1*) gene, located on chromosome 1q21.3, was identified to be responsible for DSH [3]. In the present study, we performed a mutation analysis of the *ADAR1* gene in seven Chinese families and two sporadic patients with typical DSH and identified six novel and two known mutations.

## Methods

### Ethics Statement

The current study conformed to the tenets of the Helsinki declaration and was approved by Ethical Committee of Shanghai skin disease hospital. All patients, their normal family members and 120 ethnically matched control individuals were informed about the purpose of the study and written consent was taken before recruitment and sampling. Informed written consent of minors was obtained from their guardians. Moreover, all the patients in our study gave their permission for publishing their images.

### Clinical presentations

**Fig. 1:**
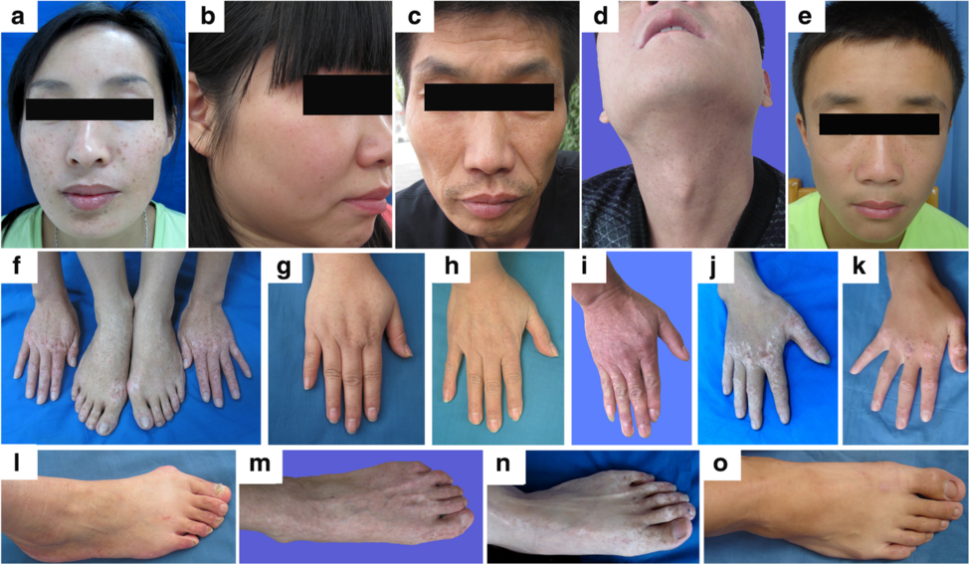
Clinical presentation of dyschromatosis symmetrica hereditaria (DSH) patients. **a.**. Freckle-like pigmented macules on the face (proband of family 3, severe phenotype).** b.** Freckle-like pigmented macules on the face of the proband in family 7. **c.** No freckle-like macules on the face of the patient II:3 in family 7. **d.** Dense freckle-like macules on the neck and face (proband of family 2, severe phenotype). **e.** Freckle-like pigmented macules on the face of the sporadic 2. **f.** Hypopigmented and hyperpigmented macules on the dorsal aspects of the extremities of hands and feet (proband of family 3, severe phenotype). **g.** Hypopigmented and hyperpigmented macules on the dorsal aspects of hands of the proband in family 7 (mild phenotype). **h.** No any lesion on the dorsal aspect of hand of the patient II:2 in family 7. **i, j.** Hypopigmented and hyperpigmented macules on the dorsal aspect of the hand (the patient II:3 in family 7 and the proband in family 2 , severe phenotype). **k.** Hypopigmented and hyperpigmented macules on the dorsal aspect of the hand of the sporadic 2. **l.** Few small freckle-like pigmented macules disturbed on the back of the foot (the patient II:2 in family 7). **m, n.** Hypopigmented and hyperpigmented macules on the back of the foot (the patient II:3 in family 7 and the proband in family 2, severe phenotype). **o.** Few small freckle-like pigmented macules disturbed on the back of the foot (the sporadic 2, mild phenotype)

This study investigated 7 families (Family 1–7) and 2 sporadic cases with DSH in the Chinese population. DSH was diagnosed by experienced dermatologists based on the typical manifestations and histopathological findings. Seven multi-generation DSH families exhibited autosomal dominant inheritance. All affected individuals had a typical mixture of hyperpigmented and hypopigmented macules on the extremities (Fig. 1a, 1f). These lesions were of irregular shapes and sizes, which appears during early childhood, generally ranging from 3 to 15 years. Table 1 summarizes the clinical findings of all the patients analyzed in this study. Noteworthy is that a variable clinical phenotype was observed in family 7. The proband in family 7 was a 23-year-old female. She presented with asymptomatic hyperpigmented and hypopigmented macules on her extremities and had been developing freckle-like macules on her face since she was 7 years old  (Figure 1b, g). Her mother has only a few small freckle-like pigmented macules disturbed on the back of her feet  (Figure 1h, l). Whereas her uncle (II:3) had many more asymptomatic hyperpigmented and hypopigmented macules on his extremities than the proband but no freckle-like macules on his face (Figure 1c, i, m). There is no systematic involvement was noticed in any of our patients.

**Table 1:** Clinical findings and predicted the effects of the mutations identified in this study

No.	Incidence	AI	UI	Party of lesions	Amid-acid substitution	Exon	Mutation type	Predicted mutation effects		
								SIFT	Polyphen 2.0	DDIG-in
1	Familial	3	7	Back of hands and feet	p.R1030DfsX1036	12	Frameshift	D	-	D
2	Familial	7	19	Back of hands and feet, ankles, forearms, neck, face	p.Stop1227R	15	Nonstop	-	-	-
3	Familial	6	11	Back of hands and feet, ankles, forearms, face	p.G803VfsX807	7	Frameshift	D	-	D
4	Familial	5	18	Back of hands and ankles	p.R360X	2	Nonsense	-	-	D
5	Familial	4	8	Back of hands and feet, face	p.D1147VfsX1184	14	Frameshift	D	-	D
6	Familial	4	6	Back of hands and feet	p.R474X	2	Nonsense	-	-	D
7	Familial	4	4	Back of hands and feet, forearms	p.R1083H	13	Missense	D	D	-
1	Sporadic	1	0	Back of hands and feet,face	p.W768X	7	Nonsense	-	-	D
2	Sporadic	1	0	Back of hands and feet, face	-	-	-	-	-	-

*AI* Affected Individuals, *UI* Unaffected Individuals, *D* Damaging; −: Not available

### Genetic studies

Polymerase chain reactions were performed as described previously [4]. In addition, samples from 120 unrelated population-match controls were sequenced for missense to exclude the possibility that these are polymorphism in the *ADAR1* gene. Mutations were identified by comparing with the reported cDNA reference sequence (GenBank accession number: NM_001111).

## Results

**Fig. 2:**
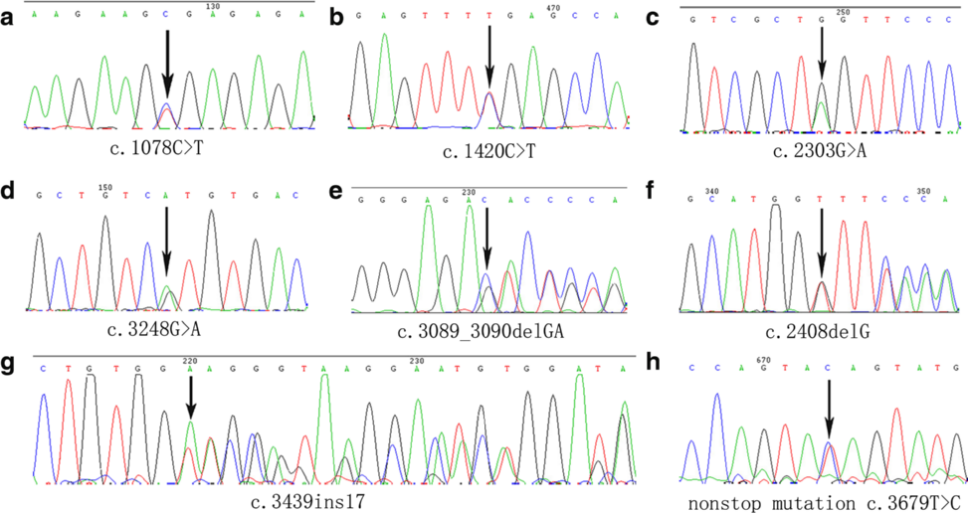
Mutations of the ADAR1 gene found in patients with dyschromatosis symmetrica hereditaria. **a.** c.1078C>T (p.R360X) mutation in family 4. **b.** c.1420C>T (p.R474X) mutation in family 6. **c.** c.2303G>A (p.W768X) mutation in Sporadic 1. **d.**c.3248G>A (p.R1083H) mutation in family 7. **e.**c.3089_3090delGA (p.R1030DfsX1036) mutation in family 1. **f.** c.2408delG (p.G803VfsX807) mutation in family 3. **g.** c.3439ins17 (p.D1147VfsX1184) mutation in family 5. **h.** a nonstop mutation TGA(Stop) CGA(Arg)=Stop1227R in family 2

The entire coding and flanking intronic sequences of *ADAR1* were screened for mutations among 7 families and 2 sporadic patients of Chinese origin with DSH, we detected six novel *ADAR1* mutations and two previously described mutations by direct sequence analysis of the PCR products (Fig. 2). The spectrum of mutations included three nonsense mutations (p.R360X, p.R474X, p.W768X), one missense mutation p.R1083H, three frameshift mutations (p.Arg1030ThrfsX1036, p.Gly803ValfsX807, p.Asp1147ValfsX1184) and one nonstop mutation p.Stop1227R. We failed to detect any *ADAR1* mutation in the sporadic 2.

Three nonsense mutations were identified in the family 4, 6 and the sporadic 1. The mutation c.1078C > T (p.R360X) was detected in exon 2 in the family 4 (Fig. 2a). The resulted truncated protein will lack 867 amino acids. The other nonsense mutation c.1420C > T (p.R474X) was detected in exon 2 in the family 6 (Fig. 2b). The predicted protein lacked 753 amino acids. In the sporadic 1, a G > A mutation was found at nucleotide 2303 that generates a translational termination codon (Fig. 2c). The predicted protein lacked 459 amino acid residues.

One missense mutation c.3248G > A was found within exon 13 and was confirmed in the other patients and excluded in the remaining unaffected persons (Fig. 2d). This transition replaced a highly conserved arginine residue with histidine in codon 1083.

Three frameshift mutations were identified in the family 1, 3 and 5. Nucleotides GA were found to delete between c.3089 and c.3090 in the family 1 and this 2-bp small deletion, designated as p.Arg1030ThrfsX1036, induced a frameshift from codon 1030 and expected to produce a premature termination codon (PTC) at codon 1036 (Fig. 2e). One single-base deletion c.2408delG was identified within exon 7 in the family 3 and generated pre-terminating codon (PTC) at 4 codons downstream of deletion site (Fig. 2f). The mutation c.3439ins17 was found within exon 14 in family 5 and this 17-bp insertion generated a PTC at 37 codons downstream of insertion site (Fig. 2g). All the above frameshift mutations generated PTC respectively, and ADAR1 protein synthesis should end there without translating the full deaminase domain, which should produce inactive enzymes of ADAR1.

Moreover, a nonstop mutation in the normal stop codon 1227 of *ADAR1* gene in exon 15 (TGA(Stop) ➙ CGA(Arg) = Stop1227R) (c.3679 T > C) was identified within exon 15 in family 2 (Fig. 2h). Theoretically, the Stop1227R mutation predictably results in an open reading frame and a mutant-type (MT) ADAR1 protein containing 1247 amino acid residues, compared with the 1226 amino acid residues of wild-type (WT) protein. The additional 21 amino acids (QYAPVTDGLGCVILGCERGRS) were included into the ADAR1 protein, with ensuing failure of formation of the healthy ADAR1 molecule and the development of DSH.

**Fig. 3:**
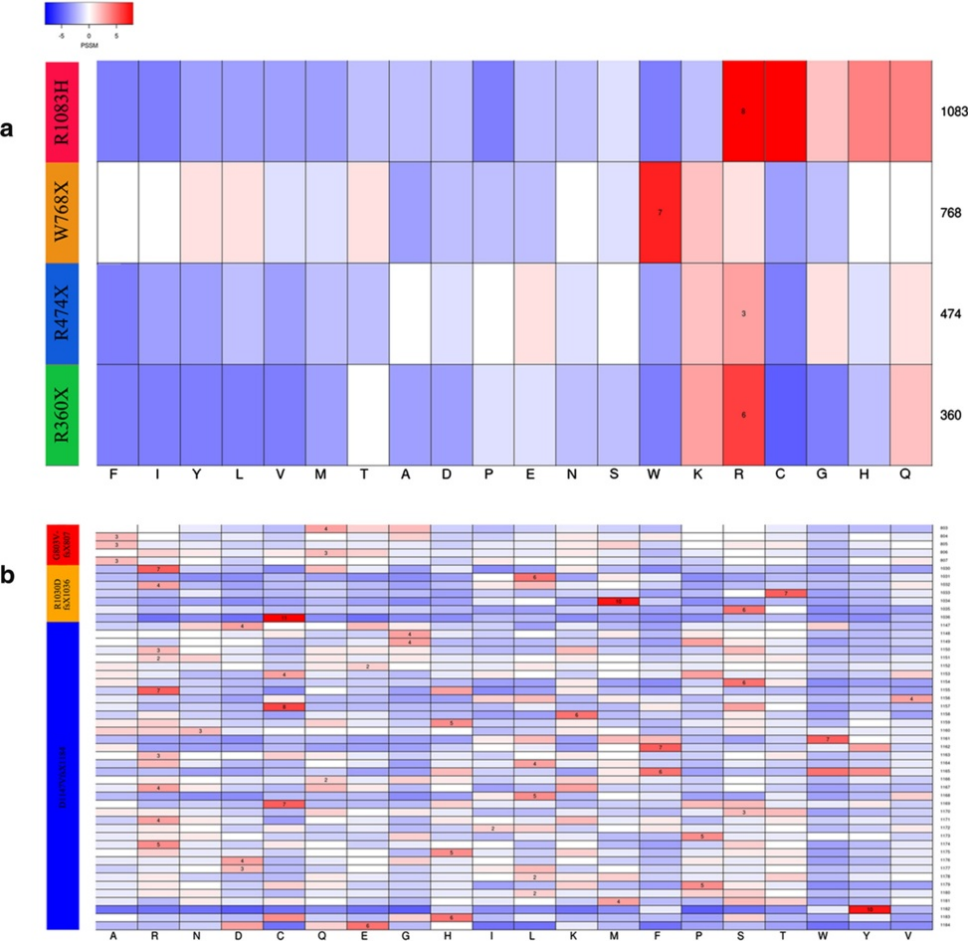
All the mutations except the nonstop mutation p.Stop1227R were predicted to be damaging. **a**. Heatmap of PSSM for single mutations (p.R360X, p.R474X, p.W768X and p.R1083H). The red color indicates more conservation while blue indicates less conservation. **b.** Heatmap of PSSM for Indels (p.G803VfsX807, p.R1030DfsX1036 and p.D1147VfsX1184). The red color indicates more conservation while blue indicates less conservation.

All the mutations except the nonstop mutation p.Stop1227R were predicted to be damaging. This prediction is based on the information from SIFT (http://sift.jcvi.org) [5], PolyPhen 2 (http://genetics.bwh.harvard.edu/pph2/) [6] and DDIG-in (http://sparks-lab.org/yueyang/server/ddig/) [7]. All the changes affect highly evolutionally conserved amino acids (Fig. 3).

## Discussion

ADAR1, also called DSRAD (double-stranded RNA-specific adenosine deaminase), belongs to a family of RNA-specific adenosine deaminase that represents one type of RNA editing enzyme. It contains at least six functional domains: two Zalpha (Z-DNA-binding domain in adenosine deaminases), three DSRM (double-stranded RNA binding motif) and a tRNA-specific and double-stranded RNA adenosine deaminase (ADEAMc) domains. They are located in exon 2, exons 2–7 and exons 9–15, respectively [8].

In this study, we detected six novel *ADAR1* mutations and two previously described mutations among 7 families and 2 sporadic patients of Chinese origin with DSH. The nonsense mutations (p.R360X, p.R474X and p.W768X) and single-base deletion c.2408delG (p.G803VfsX807) are expected to cause ADAR1 truncations lacking the entire ADEAMc domain while the other two frameshift mutations p.R1030TfsX1036 and p.D1147VfsX1184 in our study supposed to cause ADAR1 truncations lacking the partial ADEAMc domain, which should produce inactive enzymes of ADAR1 and ensue the development of this disease. All the frameshift indels were predicted to be deleterious by both SIFT and DDIG-in, all the nonsense mutations were predicted to be deleterious by DDIG-in and missense mutation c.3248G > A was predicted to be potentially damaging by both SIFT and Polyphen2.0 (Table 1). It is also possible that the mutant ADAR1 protein may not exist at all in these affected patients based on nonsense-mediated mRNA decay (NMD). NMD is a surveillance mechanism by which cells recognize and degrade mRNAs containing premature translation termination codons [9]. In family 5, we detected a nonsense mutation p.R474X, this mutation has been detected most frequently so far, and has been reported five times [3, 4, 10]. The hotspot mutation p.R474X has previously been reported in Chinese [4, 10] and also in Japanese [3] in many studies, indicating a possible founder effect of this mutation. No mutation was found in one sporadic 2 although he presents typical lesions of DSH (Fig. 1e, k, o). It is possible that the pathogenic mutation is regulatory ones within the promoter region or non coding regions. Such mutations will be missed in the current study where only the coding sequences and the exon-intron boundary sequences were targeted [4].

Of interest, we identified a nonstop mutation, c.3679 T > C (p.Stop1227R), in family 2. To our knowledge, this is a novel mutation and firstly reported in *ADAR1* gene. Previous reports found that nonstop mRNA mutations associated with Diamond-Blackfan anemia [11], mucopolysaccharidosis II [12] and FX-coagulation deficiency [13] always result in pronounced reduction of steady-state levels of mutant mRNA. In contrast, quantitative real-time PCR and 3’-RACE-RFLP analysis revealed unreduced nonstop mRNA levels in the patient with mitochondrial neurogastrointestinal encephalomyopathy harboring a nonstop mRNA mutation (c.1416delC) in the *TYMP* gene [14]. However, we could not obtain experimental evidence because we lost contact with the patients in the family and could not get an additional blood sample for the quantitative real-time PCR and 3’-RACE-RFLP analysis. The proband in this family presented with severe phenotype including dense freckle-like macules on his neck and face, and multiple dense hyper- and hypopigmented lesions on the back of his hands and feet (Fig. 1d, j, n). Further functional studies are required to elucidate the pathomechanisms of the severe DSH underlying this nonstop mutation. Although still very much speculative, the following pathomechanisms of the severe DSH underlying this mutation may be considered: i) The p.Stop1227R mutation predictably results in an open reading frame and a mutant-type (MT) ADAR1 protein containing 1247 amino acid residues, compared with the 1226 amino acid residues of wild-type (WT) protein. The additional 21 amino acids (QYAPVTDGLGCVILGCERGRS) were included into the ADAR1 protein, with ensuing failure of formation of the healthy ADAR1 molecule. ii) It is likely that the mutant mRNA of *ADAR1* gene in family 2 were reduced duing to nonstop mRNA decay, which was recently identified and showed to reduce the accumulation of abnormal transcripts lacking in-frame termination codons [11–13]. iii) The c.3679 T > C nonstop mRNA molecules are as stable as those transcribed from the wild-type alleles in this family but the translation of mutant transcript is repressed and/or its protein product is not stable [14].

Moreover, family 7 exhibits a phenotypic variability with a single germline mutation in *ADAR1*. The proband’s mother only has few small freckle-like pigmented macules disturbed on the back of her feet (Fig. 1l) whereas her uncle had much more asymptomatic hyperpigmented and hypopigmented macules on his extremities (Fig. 1i, m). According to his medical history, he used to have chilblain on his hands and feet during winter. Meanwhile, he had severe chilblain manifestated as blisters and erosions, which might have caused hypopigmented and hyperpigmented macules on his skin, which then aggravate the clinical presentation of DSH. In this family, we detected a missense mutation p.R1083H in *ADAR1* gene, this mutation has been reported in the Japanese family and its patients are early-onset (under one year old) [15], while the proband in our family had her lesions since the age of 7. To sum up, we found that the same mutation could lead to different phenotypes even in the same family and did not establish a clear correlation between genotypes and phenotypes, thereby suggesting that phenotype is not explained by genotype alone and some factors, such as viral infection in utero and/or in infancy and exposure to ultraviolet light as well as chilblain in our patient, may affect the phenotype expression [1]. Li et al. compared the clinical features with the mutation identified in all families, but they also did not find a clear correlation between genotypes and phenotypes [4].

## Conclusion

We have reported six novel mutations including a nonstop mutation p.Stop1227R, which was firstly reported in *ADAR1* gene. Furthermore, we reported a three-generation family exhibiting phenotypic variability with a single germline *ADAR1* mutation and chilblain might aggravate the clinical phenotypes of DSH. The ongoing recognition of different mutations may give insight into the still unknown mechanisms involved in the development of DSH.

## Abbreviations

ADAR1: Adenosine deaminase acting on RNA1
ADEAMc: A tRNA-specific and double-stranded RNA adenosine deaminase
DSH: Dyschromatosis symmetrica hereditaria
DSRAD: Double-stranded RNA-specific adenosine deaminase
DSRM: Double-stranded RNA binding motif
NMD: Nonsense-mediated mRNA decay
PTC: Premature termination codon
Zalpha: Z-DNA-binding domain in adenosine deaminases
